# Rapid mapping of urinary schistosomiasis: An appraisal of the diagnostic efficacy of some questionnaire-based indices among high school students in Katsina State, northwestern Nigeria

**DOI:** 10.1371/journal.pntd.0005518

**Published:** 2017-04-03

**Authors:** Tolulope Ebenezer Atalabi, Taiwo Oluwakemi Adubi, Umar Lawal

**Affiliations:** 1 Department of Biological Sciences, Faculty of Science, Federal University Dutsinma, Dutsin-Ma, Katsina State, Nigeria; 2 Department of Biological Sciences and Biotechnology, College of Pure and Applied Sciences, Caleb University, Imota, Lagos State, Nigeria; London School of Hygiene and Tropical Medicine, UNITED KINGDOM

## Abstract

**Background:**

In sub-Saharan Africa, over 200 million individuals are estimated to be infected with urinary and intestinal schistosomiasis. In a bid to lay a foundation for effective future control programme, this study was carried out with the aim of assessing the diagnostic efficacy of some questionnaire-based rapid assessment indices of urinary schistosomiasis.

**Methodology:**

A total number of 1,363 subjects were enrolled for the study. Questionnaires were administered basically in English and *Hausa* languages by trained personnel. Following informed consent, terminal urine samples were collected between 09:40 AM and 2:00 PM using clean 20 ml capacity universal bottles. 10μl of each urine residue was examined for the eggs of *S*. *haematobium* using x10 objective nose of Motic Binocular Light Microscope (China).

**Principal findings:**

The average age ± Standard Deviation (SD) of school children examined was 15.30 ± 2.30 years and 40.87% were females. The overall prevalence and geometric mean intensity of *S*. *haematobium* infection were 26.41% (24.10─28.85) and 6.59 (5.59─7.75) eggs / 10 ml of urine respectively. Interestingly, a questionnaire equivalence of the prevalence obtained in this survey was 26.41% (24.10─28.85) for Rapid Assessment Procedure based on self-reported blood in urine. The results of correlation analyses demonstrated significant associations between the prevalence of *S*. *haematobium* infection and contact with potentially infested open water sources (r = 0.741; *P* = 0.006). By regression model, cases of respondents with self-reported blood in urine are expected to rise to 24.75% if prevalence of the infection shoots up to 26.5%.

**Conclusions/Significance:**

The best RAP performance was obtained with self-reported blood in urine. Based on the overall prevalence value, the study area was at a “moderate-risk” of endemicity for urinary schistosomiasis. Chemotherapeutic intervention with Praziquantel, the rationale behind rapid assessment procedure for schistosomiasis, has been recommended to be carried out once in every 2 years for such communities.

## Author summary

Schistosomiasis is a water-borne neglected infectious disease of poverty that has consistently plagued over 200 million helpless inhabitants of the tropics, particularly, sub-Sahara Africa. Under the auspices of different nomenclatures and affiliations, many control programmes based on Praziquantel have been inaugurated over the past decades. Bearing in mind that globally, schistosomiasis exist in focal pockets within peri-urban and rural settings, and the lowest cost of a generic 600-mg tablet is approximately US$ 0.08, it is imperative to focus control resources more on high risk settings. In order to identify such settings, rapid means of mapping schistosomiasis prevalence are carried out either with questionnaires or biomedical testing with reagent strips. Rapid assessment procedure for urinary schistosomiasis, the focus of this study, builds substantially on the perception of respondents about the disease through visible blood in their urine (where applicable). We conducted this present survey in 6 communities of Katsina State, northwestern Nigeria by interviewing and examining the urine of 1, 363 high schools students for the eggs of *Schistosoma haematobium*. A unique discovery in this survey was that contact with unwholesome water bodies, where properly defined, was significantly associated with urinary schistosomiasis, both as a single index and when combined with itching experience.

## Introduction

Schistosomiasis, a water-borne neglected tropical disease (NTD), has been reported as the second most prevalent parasitic disease after malaria [1]. The causative agent of human schistosomiasis is a digenetic trematode blood fluke of the genus *Schistosoma* with a complex, indirect life cycle involving different species of freshwater snails [2, 3]. These snails serve as intermediate hosts to *S*. *haematobium*, *S*. *intercalatum*, *S*. *japonicum*, *S*. *mansoni* and *S*. *mekongi* which parasitize humans [4,5]. Humans become infected when the infective larvae mechanically penetrate their skin after contact in fresh water bodies located in environment characterized by poor hygiene and sanitation [6].

The distribution of schistosomiasisis is more abundant in the African region with 42 countries endemic for the infection. In sub-Saharan Africa, over 200 million individuals are estimated to be infected with urinary and intestinal schistosomiasis [7], with approximately 393 million people at risk of infection from *Schistosoma mansoni*, of which 54 million are infected while 436 million people are at risk of *S*. *haematobium* infection and 112 million are infected [8].

The most widely used approach for the diagnosis in endemic settings is the detection of schistosome eggs in either stool or urine specimens by light microscopy. The first step in targeting health interventions is to map the disease geographically and rank it according to the risk of infection and morbidity [4].

The use of geographical information systems highlighted the scarcity of data in endemic region such as Africa, and emphasizes the need for a rapid, non-invasive and inexpensive epidemiological assessment tool that can be fully integrated within existing administrative systems [9].

Simple school questionnaires were developed for *S*. *haematobium* and has since been validated in many ecological, epidemiological, and sociocultural settings across sub-Saharan Africa. It is well accepted and operationally feasible. It is faster and less expensive than the standard parasitological diagnosis [10]. The basis of this method was that, being a chronic disease, the only clear symptom school-age children could observe and easily remember was the presence of blood in urine [11].

They build directly on a community’s perception of disease, involve the active participation of teachers and schoolchildren, and represent a first step towards involving the community in control activities. Macrohaematuria, microhaematuria, and proteinuria are assessed by reagent strips [12]. In a bid to lay a foundation for effective future control programme for urinary schistosomiasis in the study area, we embarked on this cross-sectional survey with the aim of assessing the diagnostic efficacy of some questionnaire-based rapid assessment indices of urinary schistosomiasis.

## Methods

### Ethics statement

Written ethical clearance to conduct the survey was issued by the Ethical Committee of the Katsina State Ministry of Education, Dutsin-Ma Zonal Office. School heads and students gave oral informed consent to participate after appropriate briefing on the background and objectives of the study. Oral assent, aided by an interpreter, was provided by students after appropriate briefing on the background and objectives of the study. They demonstrated this by willingly providing their names for a written documentation during the interview. Information obtained from the subjects was kept confidential. Noteworthy is the fact that formal consent could not be obtained from the parents and guardians of the subjects partly because the cultural and religious situation of the study area was volatile. To buttress this point, there is history of attempted physical attack on healthcare officials in the study area.

### Study area

**Fig 1:**
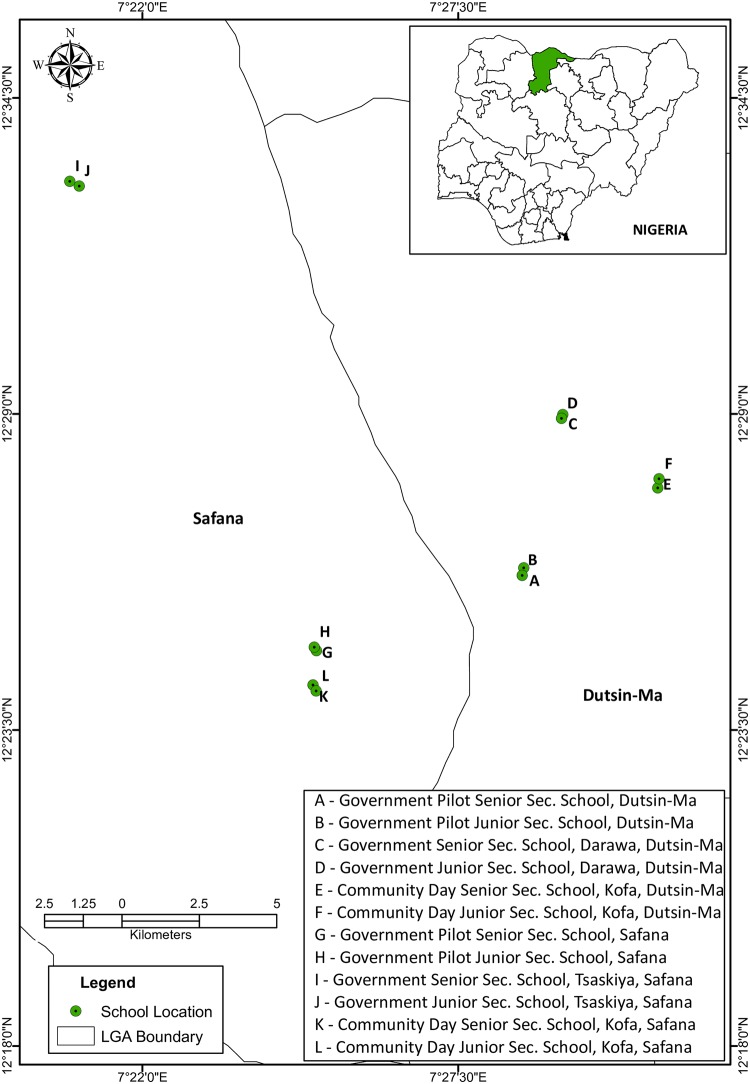
Map showing the school locations surveyed in Dutsin-Ma and Safana LGAs.

The study was undertaken in twelve (12) high schools from six (6) communities of Dutsin-Ma and Safana (809 km^2^) Local Government Areas (LGAs) of Katsina State, Northwestern Nigeria (see Fig 1). As at 2006 National Census, both neighboring LGAs were inhabited by 353,450 people [13]. Noteworthy is the fact that the study area, characteristically sandy with a rocky terrain (typical of western upland plateau) is drained by different water bodies, the largest being Zobe Dam. The study covered a bio-geographical Sudan Savannah area characterized by low to moderate endemicity for urinary schistosomiasis. By Agro-ecological classification, it belongs to the Sudan savanna vegetation zone of Nigeria [14].

The main economic activity there is farming, with millet as the subsistence crop. The predominant ethnic groups, Hausa and Fulani, complement crop production with trading and nomadism.

Both LGAs have a mean annual rainfall and temperature less than 800mm and 30°C respectively [15].

### Study design and sample size determination

A cross-sectional study design was adopted in this present survey. By estimating the prevalence of *Schistosoma haematobium* at 30% with power and sampling error of 90% and 5% respectively, a sample size of 912 was obtained. This calculation was made based on the standard of World Health Organization for sample size estimation [16]. Simple random sampling technique was employed to select the total number of 1,363 secondary school students who participated in the study between May and August, 2015. This sample size accounted for effect size and any anticipated non-response.

### Questionnaire administration

School based questionnaire with questions relating to the knowledge of urinary schistosomiasis, sources of water, and local name associated with the disease was used in the survey. For information on urinary schistosomiasis, question asked was: “Do you know any student in this school who reportedly pass blood-stained urine?” For a positive response, the next question was: “What is the local language for this condition?” The individual questionnaire was designed, among other things, to obtain responses from subjects on water contact activities (fetching, swimming and play in shallow water), experiences of itching, haematuria, and pain while urinating. To elicit responses for some of these experiences, interviewees were asked the following questions: “Have you ever experienced: (i) pains while urinating? (ii) blood in your urine?”

Whenever a response was positive for the latter, each subject was further questioned: “How have you been treating it?”

Because the study population was divided along the language lines of English and *Hausa*, questionnaires were administered accordingly by trained personnel which included the investigators and selected teachers from the participating schools.

### Parasitological techniques

#### Urine collection and processing

Following informed consent, interviewees were subjected to a parasitological procedure which commenced with the collection of terminal urine samples using clean 20 ml capacity universal bottles between 9:40 AM and 2:00 PM [17]. Biochemical parameters of urine such as haematuria, proteinuria, bilirubin, urobilinogen, pH, specific gravity, ketone, glucose, nitrite and leucocytes were tested using urine reagent strips (Combi 10; UriScreen, German Technology) shortly after sample collection.

About 5ml of each sample was centrifuged at 2,250 r.p.m for 1 ½ minutes using Centurion Scientific Centrifuge (C2 series) made in United Kingdom.

### Microscopy and identification of *S*. *haematobium* eggs

In the study area, *S*. *mansoni* is co-endemic with *S*. *haematobium*. Consequently, some interviewees suffered a mixed infection. Consequently, there was unusual discovery of the eggs of the former (*S*. *mansoni*) in a few urine samples that were as well positive for the eggs of the latter (*S*. *haematobium*). They were distinguished using their unique identification keys, that is, the possession of a lateral and terminal spine by the eggs of *S*. *mansoni* and *S*. *haematobium* respectively [18]. However, it has been reported that under special epidemiological settings with a very high prevalence of urinary schistosomiasis but a very low prevalence of intestinal schistosomiasis, eggs of *S*. *mansoni* do occur in urine [19].

10μl of each urine residue was examined for the eggs of *S*. *haematobium* using x10 objective nose of Motic Binocular Light Microscope (China). Each average egg count was recorded as number of eggs per 10 ml of urine sample using a multiplier factor of two. While prevalence was grouped into low (˂ 10%), moderate (≥ 10%–49%) and high (≥ 50% or more) [20], intensity of infection was categorized into light (˂ 50 eggs / 10 ml of urine) and heavy (≥ 50 eggs / 10 ml of urine) infections according to standard method [4].

### Data analysis

**Fig 2:**
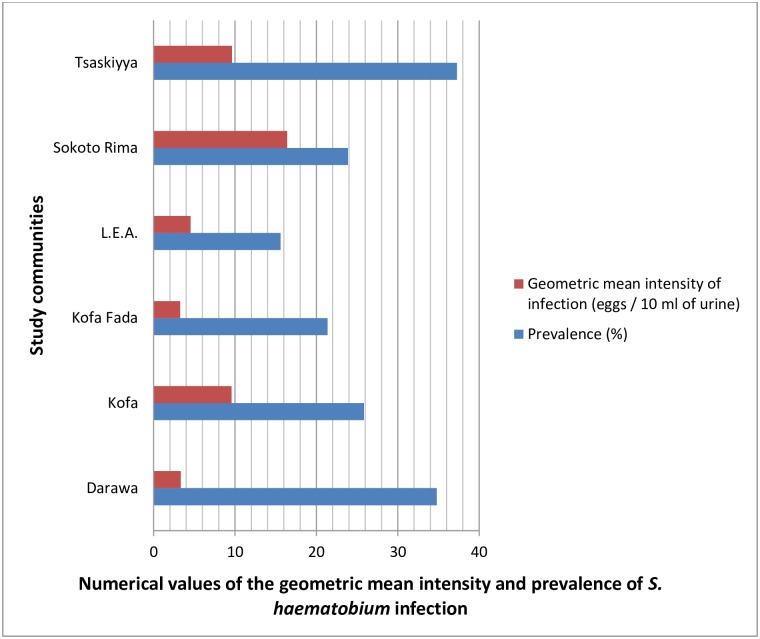
Chart showing the prevalence and intensity of urinary schistosomiasis by study community.

**Fig 3:**
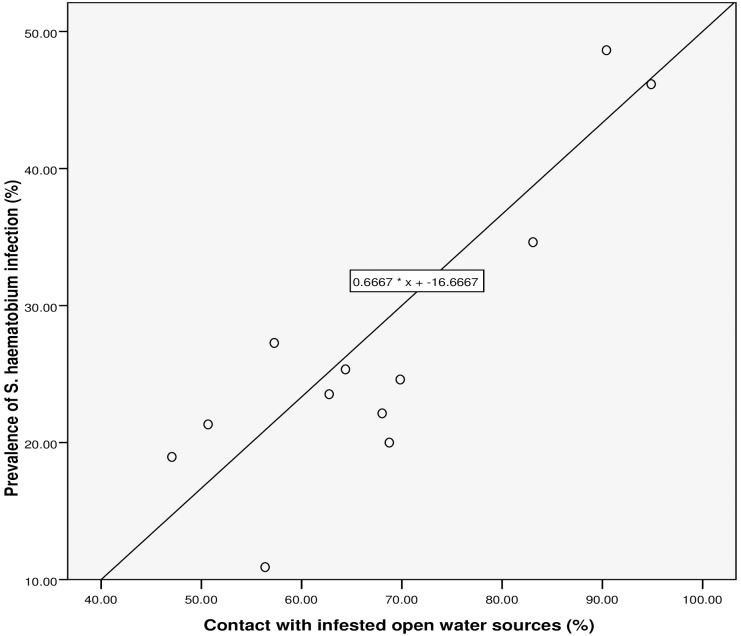
Relationship between prevalence of *S*. *haematobium* infection and RAP based on contact with infested open water sources.

**Fig 4:**
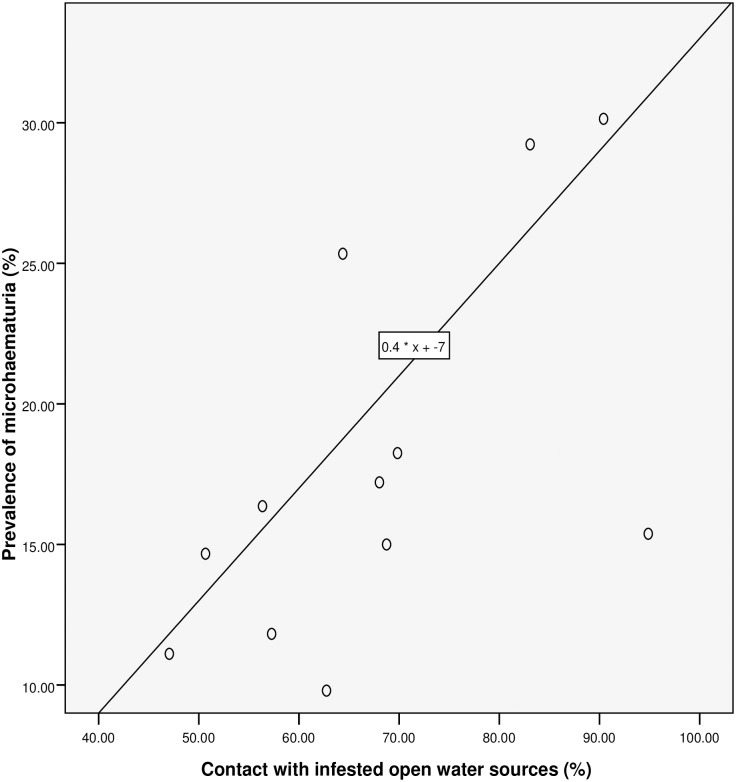
Relationship between prevalence of microhaematuria and RAP based on contact with infested open water sources.

**Fig 5:**
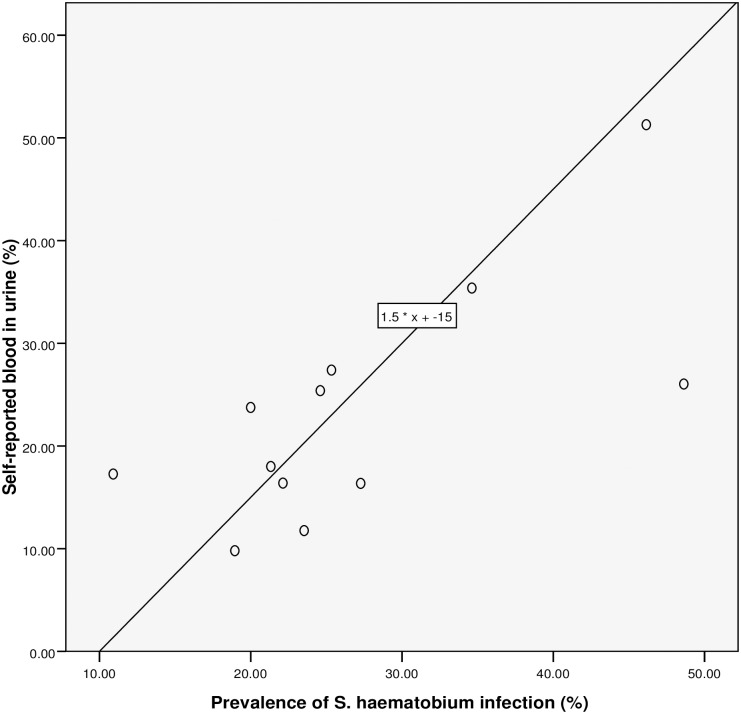
Relationship between RAP based on self-reported blood in urine and prevalence of *S*. *haematobium* infection.

**Fig 6:**
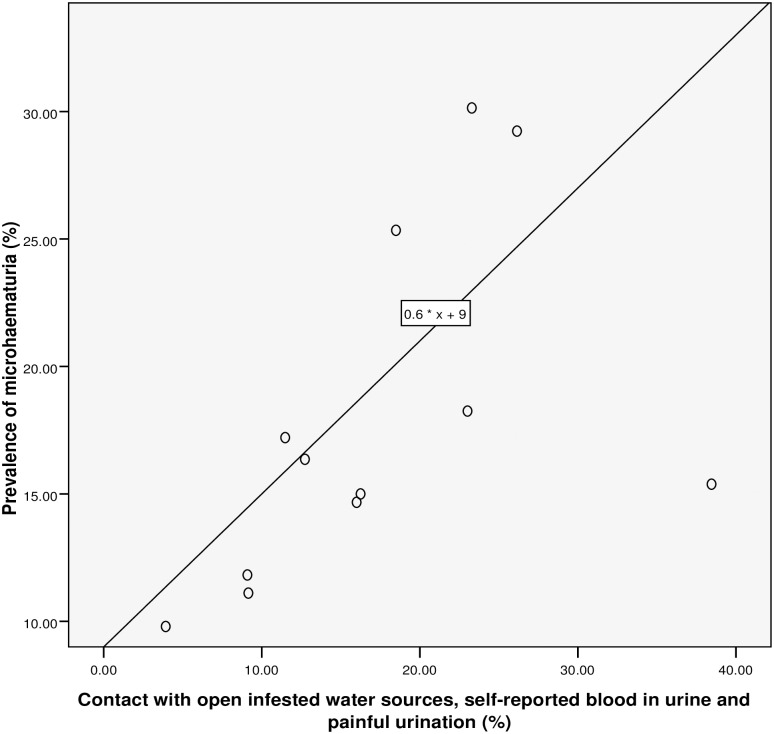
Relationship between microhaematuria and combined RAP based on contact with infested water sources, self-reported blood in urine and painful urination.

**Fig 7:**
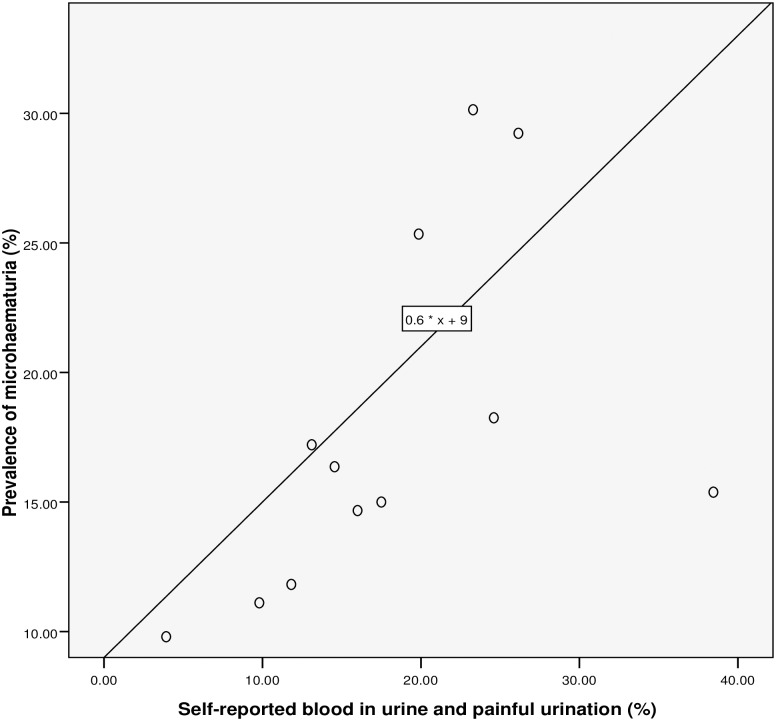
Relationship between microhaematuria and combined RAP based on self-reported blood in urine and painful urination.

All data obtained from the survey were entered into Microsoft Excel 2010 (USA) and analysed using SPSS 15.0 (Chicago, USA). The relationships between Rapid Assessment and Parasitological Indices were assessed using Spearman’s rank correlation and linear regression analyses. The prevalence for *S*. *haematobium* infection, as well as for micro-haematuria (shown in Figs 2–7) was calculated on a school-level basis. These school-level estimates were correlated with the following rapid assessment indicators [i.e. water contact (in %), self-reported blood in urine (in %), itching, urethral pain, and combined RAPs based on water contact/ self-reported blood in urine/ itching/ urethral pain (in %), and water contact/ itching (in %)] using the Spearman's rank correlation coefficient test. Both school-level prevalences of *S*. *haematobium* infection and micro-haematuria were used in a linear regression model as continuous dependent outcomes (in %) and were controlled for, in a univariable manner, each rapid assessment indicator. Prior to reporting our findings, necessary diagnostic tests were performed. Normality was achieved and no key model assumptions were violated. Statistical significance was considered at 95% confidence level (CL) with a *P* value of 0.05.

The diagnostic performances of indices for identifying “low risk”, “moderate risk” or “high risk” schools were assessed by calculating sensitivities, specificities, and positive and negative predictive values.

## Results

### Characteristics of the study population

In the six (6) communities surveyed, 1,363 students with ages ranging from 10–25 years were interviewed and examined. The average age ± Standard Deviation (SD) of school children examined was 15.30 ± 2.30 years and 40.87% were females.

### Prevalence and intensity of *S*. *haematobium* infection

Of the total number interviewed, 360 respondents were infected. It is worthy of note that the prevalence and geometric mean of *S*. *haematobium* egg counts in the study communities ranged from 15.59% to 37.28% and 3.27 to 16.42 eggs / 10 ml of urine respectively (see Table 1 and Fig 2). Hence, the overall prevalence and geometric mean intensity of *S*. *haematobium* infection were 26.41% (24.10–28.85) and 6.59 (5.59–7.75) eggs / 10 ml of urine respectively. The arithmetic mean intensity of infection was 27.90 (19.55–36.25) eggs / 10 ml of urine.

**Table 1:** Prevalence and intensity of urinary schistosomiasis by study location and sex.

Communities	N.S	N.I	Prevalence (%) (95% Confidence Interval)	Geometric Mean intensity of infection (95% Confidence Interval)	Crude Odds Ratio (95% Confidence Interval)
**Darawa**	296	103	34.80 (29.38–40.52)	3.34 (2.63–4.24)	2.89 (1.92–4.35)
**Kofa**	236	61	25.85 (20.39–31.93)	9.59 (6.05–15.23)	1.89 (1.21–2.94)
**Kofa Fada**	131	28	21.37 (14.70–29.39)	3.27 (1.99–5.36)	1.47 (0.86–2.51)
**L.E.A.**	263	41	15.59 (11.43–20.55)	4.53 (3.09–6.65)	1 (reference)
**Sokoto Rima**	268	64	23.88 (18.90–29.45)	16.42 (11.10–24.28)	1.69 (1.09–2.63)
**Tsaskiyya**	169	63	37.28 (29.97–45.04)	9.63 (6.55–14.16)	3.22 (2.04–5.08)
**Total**	**1003**	**360**			
***χ***^**2**^					0.00164
***P value***					0.9677
**Sex**					
**Male**	806	323	40.07 (36.69–43.56)	7.52 (6.33–8.94)	9.39 (6.54–13.49)
**Female**	557	37	6.64 (4.78–9.12)	2.08 (1.54–2.80)	1 (reference)
**Overall**	**1003**	**360**	**26.41 (24.10–28.85)**	**6.59 (5.59–7.75)**	
***χ***^**2**^					189.269
***P value***					˂ 0.0001

Abbreviation: NS, Number surveyed; NI, Number infected.

Males recorded a higher prevalence [40.07% (36.69–43.56)] and geometric mean intensity of *S*. *haematobium* infection [7.52 (6.33–8.94) eggs / 10 ml of urine].

Furthermore, males were 9 times [COR (95% CI): 9.39 (6.54–13.49)] more likely to be infected with the cercariae of *S*. *haematobium* (see Table 1).

### Performance of the questionnaire

Spearman’s rank correlation (see Table 2 and Figs 3–7) demonstrated significant associations between: *S*. *haematobium* prevalence and contact with potentially infested open water sources (r = 0.741; *P* = 0.006); prevalence of micro-haematuria and contact with potentially infested open water sources (r = 0.643; *P* = 0.024); self-reported blood in urine and prevalence of *S*. *haematobium* infection (r = 0.629; *P* = 0.028); prevalence of micro-haematuria and the combined RAP of water contact, self-reported blood in urine and painful urination (r = 0.741; *P* = 0.006) and prevalence of micro-haematuria and the combined RAP of self-reported blood in urine and painful urination (r = 0.727; *P* = 0.007). Significant associations (see Table 2) were also obtained with: prevalence of *S*. *haematobium* infection and the combined RAP of water contact, and itching (r = 0.636; *P* = 0.026); and prevalence of *S*. *haematobium* infection and the combined RAP of self-reported blood in urine, and painful urination (r = 0.587; *P* = 0.045). The questionnaire equivalence of the prevalence revealed a similar prevalence of 26.41% (24.10–28.85) for RAP based on self-reported blood in urine (see Fig 5).

**Table 2:** Spearman correlation coefficients for the relationship between rapid assessment indices and parasitological indicators of *S*. *haematobium* infection.

Rapid assessment indicator	Parasitological indicator of *S*. *haematobium* endemicity				
	Prevalence	Prevalence of light infection	Prevalence of heavy infection	SCEL	Prevalence of micro-haematuria
**Water contact (%)**	0.741 (0.006) **	0.573 (0.051)	0.577 (0.050) **	0.364 (0.245)	0.643 (0.024) **
**SRBU (%)**	0.629 (0.028) **	0.357 (0.255)	0.676 (0.016) **	0.490 (0.106)	0.734 (0.007) **
**Itching (%)**	0.608 (0.036) **	0.650 (0.022) **	0.441 (0.151)	0.378 (0.226)	0.315 (0.319)
**Water contact + SRBU+ Pain (%)**	0.573 (0.051)	0.392 (0.208)	0.573 (0.051)	0.364 (0.245)	0.741 (0.006) **
**Water contact + Itching (%)**	0.636 (0.026) **	0.615 (0.033) **	0.502 (0.096)	0.434 (0.159)	0.357 (0.255)
**SRBU + Pain (%)**	0.587 (0.045) **	0.378 (0.226)	0.634 (0.027) **	0.469 (0.124)	0.727 (0.007) **
**Pain (%)**	0.140 (0.665)	0.000 (0.100)	0.217 (0.498)	-0.007 (0.983)	0.538 (0.071)

**Statistically significant association (*P* ≤ 0.05). Number in bracket is the *P*-value while the one outside is the coefficient of Spearman’s correlation. SCEL, School community egg load; SRBU, Self-reported blood in urine.

However, no significant association was recorded between the prevalence of light infection intensity and water contact (r = 0.573; *P* = 0.051); school community egg load and water contact (r = 0.364; *P* = 0.245); prevalence of micro-haematuria and the combined RAP of water contact, and itching (r = 0.357; *P* = 0.255). Noteworthy was the fact that the single RAP index of pain while urinating recorded the poorest performance since it had no statistically significant association (*P* ˃ 0.05) with any Parasitological indices (see Table 2).

### The linear regression model

We discovered that, for a 26.5% rise in contact with infested water sources, the: prevalence of *S*. *haematobium* infection will increase by 1% (using the equation: y = 0.6667 * x– 16.6667 shown in Fig 3); prevalence of microhaematuria will only increase by 3.6% (see Fig 4). However, cases of respondents with self-reported blood in urine are expected to rise to 24.75% if prevalence of the infection shoots up to 26.5% (see Fig 5). Meanwhile, using equation 0.6 * x + 9, the prevalence of microhaematuria is anticipated to increase by 24.9% with a similar 26.5% rise in the number of respondents with combined experiences of contact with infested water sources, self-reported blood in urine and painful urination (see Fig 6). Similarly, for 26.5% increase in the number of respondents with combined experiences of contact with infested water sources and self-reported blood in urine, the prevalence of microhaematuria is expected to remain 24.9% (see Fig 7).

### Agreement between techniques

All RAPs showed sensitivities which ranged from 33.33–98.08%. However, RAPs based on water contact, pain while urinating and self-reported blood in urine respectively recorded high sensitivities in descending order of magnitude. Meanwhile, specificity ranged from 45.46–94.32%. The least value was recorded for RAP based on water contact while the highest was obtained with the combined RAP of water contact, self-reported blood in urine and pain while urinating.

In summary, the best RAP performance was obtained with self-reported blood in urine which had a sensitivity of 60.28% and specificity of 91.43%. Co-incidentally, this RAP also recorded the best combined values for Positive Predictive Value (PPV) and Negative Predictive Value (NPV) (Table 3).

**Table 3:** Sensitivity, specificity, positive predictive value and negative predictive value of the different RAP indices.

	Urine microscopy					
RAP indices	Negative	Positive	Sensitivity (%)	Specificity (%)	PPV (%)	NPV (%)
**Water contact**						
**Negative**	456^c^	7^b^	98.06	45.46	39.22	98.49
**Positive**	547^d^	353^a^				
**Total**	**1003**	**360**				
**Self-reported blood in urine (SRBU)**						
**Negative**	917	143	60.28	91.43	71.62	86.51
**Positive**	86	217				
**Total**	**1003**	**360**				
**Itching**						
**Negative**	870	239	33.61	86.74	47.64	78.45
**Positive**	133	121				
**Total**	**1003**	**360**				
**Pain while urinating**						
**Negative**	738	135	62.50	73.58	45.92	84.54
**Positive**	265	225				
**Total**	**1003**	**360**				
**Water contact + SRBU + Pain**						
**Negative**	946	183	49.17	94.32	75.64	83.79
**Positive**	57	177				
**Total**	**1003**	**360**				
**Water contact + Itching**						
**Negative**	873^c^	240^b^	33.33	87.04	48.00	78.09
**Positive**	130^d^	120^a^				
**Total**	**1003**	**360**				

^PPV, Positive predictive value; NPV, Negative predictive value; SRBU, Self-reported blood in urine;^

^a^ = True positive;

^b^ = False negative;

^c^ = True negative;

^d^ = False positive.

## Discussion

Currently, the most widely used clinical approach to determining the prevalence and intensity of infection due to *S*. *haematobium* is manual egg count by means of urine microscopy. Our data showed that by this gold standard [17, 21], the overall prevalence and geometric mean intensity of urinary schistosomiasis were 26.41% (24.10–28.85) and 6.59 (5.59–7.75) eggs / 10 ml of urine respectively, with males being 9 times [COR (95% CI): 9.39 (6.54–13.49)] more likely to be infected compared to females.

It is pertinent to state that this gold standard employed *vis a vis* the rapid assessment procedures in this cross-sectional survey was rather cumbersome and frustrating. However, it is of interest that a questionnaire equivalence of the prevalence obtained in this survey revealed a similar prevalence of 26.41% (24.10–28.85) for RAP based on self-reported blood in urine. Again, findings in this survey showed that the best RAP performance was obtained with self-reported blood in urine with a sensitivity and specificity of 60.28% and 91.43% respectively.

To corroborate the reliability of this RAP index, a report from Yemen shows that 72.2% of respondents who suffered heavy intensity of infection with *Schistosoma haematobium* visibly experienced blood in their urine [17].

Furthermore, high sensitivity and specificity have previously been reported in other urinary schistosomiasis endemic settings. For example, in a similar survey conducted in southwestern Nigeria, a specificity of almost 100% was obtained [22]. In northern Ghana, self-reported haematuria showed a sensitivity of 53% and a specificity of 85% [23].

Co-incidentally, in this survey, self-reported blood in urine also recorded the best results for Positive Predictive Value (71.62%) and Negative Predictive Value (86.51%). The latter simply means that, of all the subjects who tested negative for urinary schistosomiasis by microscopic examination, 86.51% were actually negative while 13.49% were positive, going by questionnaire-based rapid means of assessment using self-reported blood in urine (macro-haematuria). In addition, when this RAP was combined with water contact and pain while urinating (dysuria), a higher Positive Predictive Value (75.64%) was obtained. That is, this combination unmasked 4.02% of more subjects that were infected compared to the single RAP. This is indeed a cost-effective means of improving on the quality of data obtained in urinary schistosomiasis research.

Moreover, the result of correlation analysis demonstrated a statistically significant association between self-reported blood in urine and prevalence of *S*. *haematobium* infection (r = 0.629; *P* = 0.028). Better still, when self-reported blood in urine was combined with RAP based on painful urination, a stronger association (r = 0.727; *P* = 0.007) was obtained between them and micro-haematuria.

Meanwhile self-reported blood in urine, micro-haematuria and painful urination (dysuria) have been previously identified as morbidity markers of urinary schistosomiasis [17, 24, 25]. The implication of these is that we can use a RAP based on self-reported blood in urine to predict the parasitological prevalence of urinary schistosomiasis in either moderate or high endemic settings. It could as well produce a reliable result in areas where biomedical reagent strips are not available [26].

Previous studies carried out in some African countries (Cameroon, Congo, Democratic Republic of the Congo, Ethiopia, Malawi, Zambia and Zimbabwe) also showed that macro-haematuria had a very good diagnostic ability to detect “high-risk” schools while ruling out “low-risk” ones [10].

In a survey conducted in the Tanga region of the United Republic of Tanzania, average of 75% school-age children were reportedly accurate in their self—diagnosis of urinary schistosomiasis using the presence of blood in urine (haematuria) as a rapid diagnostic procedure [27].

To the best of our knowledge, contact with potentially infested, open, and unwholesome water sources is not in use as a rapid assessment indicator for urinary schistosomiasis. However, in this survey, the result of correlation analysis demonstrated significant association between prevalence of *S*. *haematobium* infection and contact with potentially infested open water sources (r = 0.741; *P* = 0.006). This did not come as a surprise because urinary schistosomiasis has been constantly reported as a water-borne disease [10, 22, 28].

When employed as a single RAP index in this present survey, it recorded a very high sensitivity (98.06%) and Negative Predictive Value (98.49%) but low values for the duo of specificity (45.46%) and Positive Predictive Value (39.22%). Meanwhile, when combined with self-reported blood in urine and dysuria, its sensitivity markedly reduced to almost half (49.17%) while its Negative Predictive Value dropped to 83.79%. Its specificity (94.32%) and Positive Predictive Value (75.64%), however, approximately doubled. It also demonstrated a statistically significant association (r = 0.643; *P* = 0.024) with the prevalence of micro-haematuria. More interestingly, when combined with other RAPs based on self-reported blood in urine and painful urination, a stronger relationship was obtained with the prevalence of micro-haematuria (r = 0.741; *P* = 0.006). The implication of these findings is that when subjects are carefully interviewed as regards their water contact activities, to a large extent, a good rapid diagnostic result for urinary schistosomiasis could be obtained.

This is a good news to all high risk endemic settings where diagnostic kits and microscopes are very short in supply. Bearing in mind that indiscriminate mass chemotherapeutic intervention with Praziquantel is indeed not harmful [27], on the basis of this finding, it could be achieved successfully without anticipating any severe adverse reactions. In the context of Schistosomiasis Elimination Strategy and Potential Role of a Vaccine in Achieving Global Health Goals co-sponsored by Bill and Melinda Gates Foundation and the National Institute of Allergy and Infectious Diseases [29], self-reported blood in urine, as a single or combined RAP index, could play a major diagnostic role in unraveling new endemic foci for mass drug administration. As it stands, self-reported blood in urine will continue to be a relevant rapid diagnostic RAP index until schistosomiasis is eradicated.

This survey is, however, subject to some limitations. To start with, adults were not included. Therefore, the result reported here may not be applicable to the whole population of the study area because previous findings have shown that the diagnostic efficacy of haematuria as a RAP index is inversely proportional to the age of subjects but stable in teenage children [30].

An extrapolation is only applicable after a painstaking assessment of the school enrollment, and the overall socio-cultural and epidemiological condition of the study area [11].

Moreover, previous report has shown that the accuracy of macro-haematuria as a yardstick for rapid assessment of urinary schistosomiasis may be better when a day-to-day variation in eggs excretion is considered [31]. However, this survey did not capture a serial assessment of each subject for macro-haematuria.

Since report has shown that the identification of schools and communities endemic for schistosomiasis is a key issue in any control programme [32], the high performing RAPs in this study could be employed to discover more endemic foci in the existence of ongoing regular Mass Drug Administration (MDA) in Nigeria.

## Conclusion

Based on this overall prevalence value of 26.41% (24.10–28.85) obtained in this survey, it is obvious that the study area was at a “moderate-risk” of endemicity for urinary schistosomiasis [4, 20]. Meanwhile, chemotherapeutic intervention with Praziquantel, the rationale behind rapid assessment procedure for schistosomiasis, has been recommended to be carried out once in every 2 years for such communities [20]. Although, water contact was found to have a good diagnostic efficacy, The best RAP in this survey was self-reported blood in urine. Both RAPs performed better when combined with other RAP indices.

## Supporting information

S1 File STROBE checklist. (DOC)

S2 File Ethical clearance. (DOCX)

S1 Table Geographical coordinates, prevalence and mean intensity by study location. (XLSX)
