# Supplementary material for: Rapid mapping of urinary schistosomiasis: An appraisal of the diagnostic efficacy of some questionnaire-based indices among high school students in Katsina State, northwestern Nigeria
Source: PLoS Negl Trop Dis. 2017 Apr 3;11(4):e0005518. doi: 10.1371/journal.pntd.0005518 (PMC5391124; doi:10.1371/journal.pntd.0005518)
Supplement: S1 File — (DOC) [file pntd.0005518.s001.doc]

STROBE Statement—Checklist of items that should be included in reports of ***cross-sectional studies***

|  | Item No | Recommendation |
| --- | --- | --- |
| **Title and abstract** | 1 | (*a*) Indicate the study’s design with a commonly used term in the title or the abstract  The title “Rapid Mapping of Urinary Schistosomiasis: An Appraisal of Some Questionnaire-Based Indices Using Parasitological Indicators Among High School Students in Katsina State, Northwestern Nigeria” is devoid of uncommonly used terms. |
|  |  | (*b*) Provide in the abstract an informative and balanced summary of what was done and what was found  Refer to lines 18-43 of the manuscript. The abstract reflects a summary of the key methodology and findings. It is self-explanatory and comprehensible. |
| Introduction | | |
| Background/rationale | 2 | Explain the scientific background and rationale for the investigation being reported  The introduction to the manuscript starts with a broad knowledge of schistosomiasis and narrows down to relevant information on the issue investigated (see lines 61-95 on pages 4 & 5). |
| Objectives | 3 | State specific objectives, including any prespecified hypotheses  The objective of the study was to assess the diagnostic efficacy of some questionnaire-based rapid assessment indices of urinary schistosomiasis (see lines 93-95, page 5 of manuscript). No prespecified hypotheses declared. |
| Methods | | |
| Study design | 4 | Present key elements of study design early in the paper  The key elements of study design have been presented on pages 6 & 7 of the manuscript. |
| Setting | 5 | Describe the setting, locations, and relevant dates, including periods of recruitment, exposure, follow-up, and data collection  Refer to lines 110-113, page 6 of the manuscript for the information on the setting, locations including date of recruitment. |
| Participants | 6 | (*a*) Give the eligibility criteria, and the sources and methods of selection of participants  The study was designed to target high school students. Simple random sampling technique was employed to select the subjects who participated in the study. |
| Variables | 7 | Clearly define all outcomes, exposures, predictors, potential confounders, and effect modifiers. Give diagnostic criteria, if applicable  In this present survey, prevalence and mean intensity were outcome variables while exposure variables included study location and sex. Micro- & macro-haematuria were potential confounders. |
| Data sources/ measurement | 8* | For each variable of interest, give sources of data and details of methods of assessment (measurement). Describe comparability of assessment methods if there is more than one group  All data from this survey were obtained from secondary school students using well-structured individual questionnaire capturing sex, age, socio-economic factors, experience of blood in urine, and so on. School community-based questionnaire with questions relating to the knowledge of urinary schistosomiasis, sources of water, and local name associated with the disease was used in the survey. |
| Bias | 9 | Describe any efforts to address potential sources of bias  To rule out bias, simple random sampling technique was employed to select the subjects who participated in the study. |
| Study size | 10 | The sample size was determined according to the standard of World Health Organization for sample size estimation. By estimating the prevalence of *Schistosoma haematobium* at 30% with power and sampling error of 90% and 5% respectively, a sample size of 912 was obtained. A total number of 1,363 subjects enrolled in the study, however, accounted for effect size and any anticipated non-response. |
| Quantitative variables | 11 | Explain how quantitative variables were handled in the analyses. If applicable, describe which groupings were chosen and why  Quantitative variables were expressed as: % (prevalence), average number of eggs/ 10ml of urine (mean intensity). No variable grouping. |
| Statistical methods | 12 | (*a*) Describe all statistical methods, including those used to control for confounding  All statistical methods used in the survey are clearly stated in lines 158-174 on pages 8 & 9 of the manuscript. However, control for confounding factors does not apply to this study. |
|  |  | (*b*) Describe any methods used to examine subgroups and interactions  Lineal regression was used to analyse the relationship between variables. Diagnostic test was run to test for compliance with the assumption of normalcy. |
|  |  | (*c*) Explain how missing data were addressed. **Not applicable.** |
|  |  | (*d*) If applicable, describe analytical methods taking account of sampling strategy  **Not applicable.** |
|  |  | (*e*) Describe any sensitivity analyses  Sensitivity was calculated by dividing true positive value (a) by the sum of the values for true positive and false negative (b). |
| Results | | |
| Participants | 13* | (a) Report numbers of individuals at each stage of study—eg numbers potentially eligible, examined for eligibility, confirmed eligible, included in the study, completing follow-up, and analysed  1, 363 individuals were confirmed eligible, included in the study, and analysed |
|  |  | (b) Give reasons for non-participation at each stage  Not applicable |
|  |  | (c) Consider use of a flow diagram  This may not be feasible for this study because of its peculiarities i.e if one is produced, its outlook might not warrant inclusion in the manuscript. |
| Descriptive data | 14* | (a) Give characteristics of study participants (eg demographic, clinical, social) and information on exposures and potential confounders  Ages of participants ranged from 10─25 years. The average age ± Standard Deviation (SD) of school children examined was 15.30 ± 2.30 years and 40.87 % were females (see lines 188-190 in the manuscript). |
|  |  | (b) Indicate number of participants with missing data for each variable of interest  Not applicable |
| Outcome data | 15* | Report numbers of outcome events or summary measures  Not applicable |
| Main results | 16 | (*a*) Give unadjusted estimates and, if applicable, confounder-adjusted estimates and their precision (eg, 95% confidence interval). Make clear which confounders were adjusted for and why they were included  Refer to Table 1on page 11 in the manuscript where some data are shown with their confidence intervals. Confounders were not adjusted for in the study. |
|  |  | (*b*) Report category boundaries when continuous variables were categorized  **Not applicable.** |
|  |  | (*c*) If relevant, consider translating estimates of relative risk into absolute risk for a meaningful time period  **Not applicable.** |
| Other analyses | 17 | Report other analyses done—eg analyses of subgroups and interactions, and sensitivity analyses  These have been addressed above. |
| Discussion | | |
| Key results | 18 | Summarise key results with reference to study objectives  Key results were summarised in lines 264-268, page 15 of the manuscript. |
| Limitations | 19 | Discuss limitations of the study, taking into account sources of potential bias or imprecision. Discuss both direction and magnitude of any potential bias.  Lines 331-340 on page 18 of the manuscript address the limitations of this present study. |
| Interpretation | 20 | Give a cautious overall interpretation of results considering objectives, limitations, multiplicity of analyses, results from similar studies, and other relevant evidence  Interpretations of results from this survey, including references to similar findings from other places as well as limitations of the study have been addressed in lines 270-340 of the manuscript. |
| Generalisability | 21 | Discuss the generalisability (external validity) of the study results  The external validity/applicability of this study has been addressed in lines 341-344, page 18 of the manuscript. |
| Other information | | |
| Funding | 22 | Give the source of funding and the role of the funders for the present study and, if applicable, for the original study on which the present article is based  This is not applicable to this study because it was self-funded. A statement on this has been included in the manuscript (see lines 364 & 365 on page 19). |

*Give information separately for exposed and unexposed groups.

**Note:** An Explanation and Elaboration article discusses each checklist item and gives methodological background and published examples of transparent reporting. The STROBE checklist is best used in conjunction with this article (freely available on the Web sites of PLoS Medicine at http://www.plosmedicine.org/, Annals of Internal Medicine at http://www.annals.org/, and Epidemiology at http://www.epidem.com/). Information on the STROBE Initiative is available at www.strobe-statement.org.
