# Supplementary figures and images for: Rapid mapping of urinary schistosomiasis: An appraisal of the diagnostic efficacy of some questionnaire-based indices among high school students in Katsina State, northwestern Nigeria
Source: PLoS Negl Trop Dis. 2017 Apr 3;11(4):e0005518. doi: 10.1371/journal.pntd.0005518 (PMC5391124; doi:10.1371/journal.pntd.0005518)

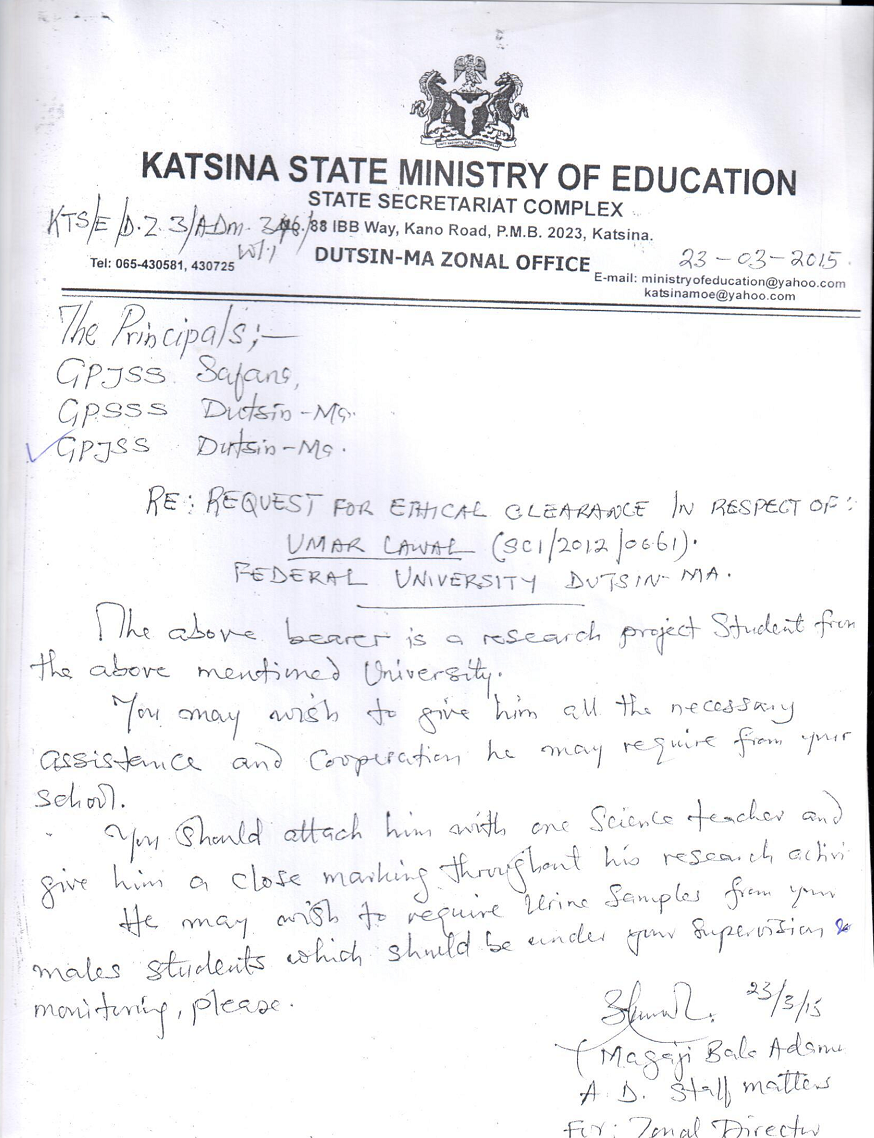


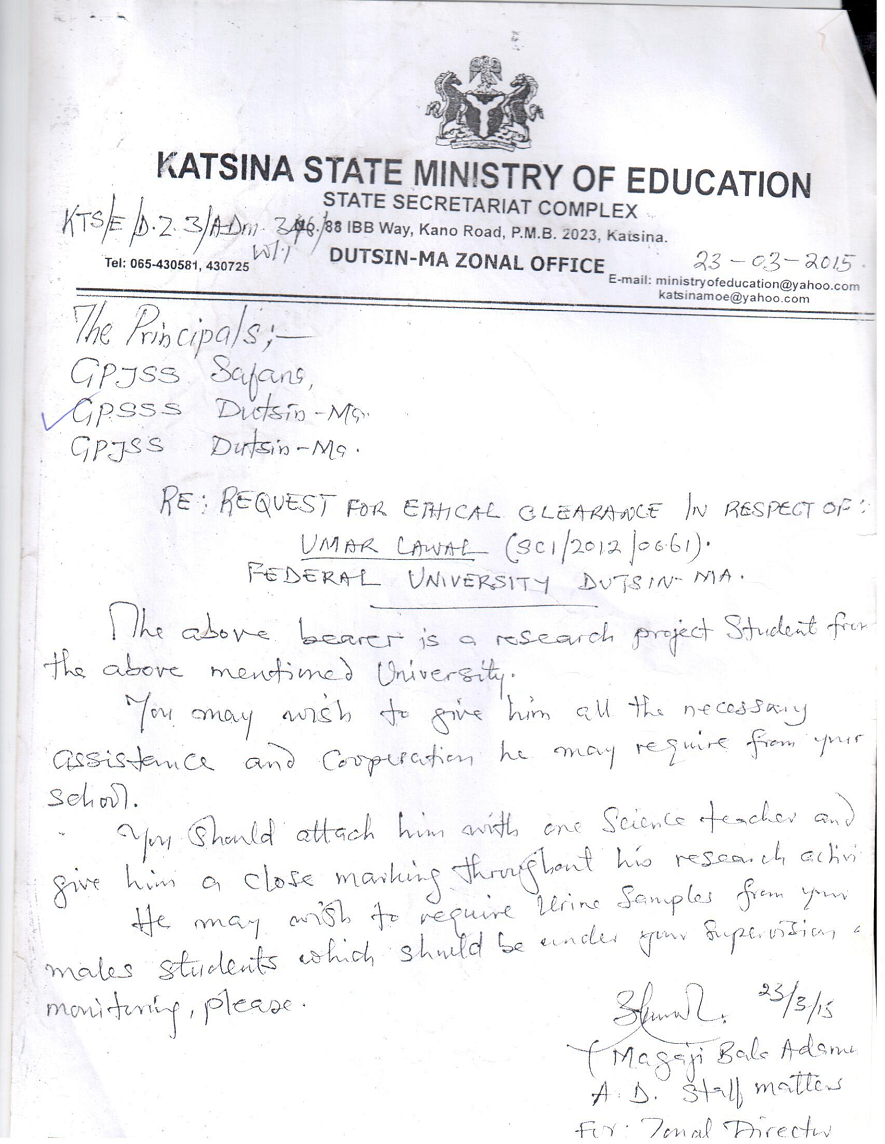


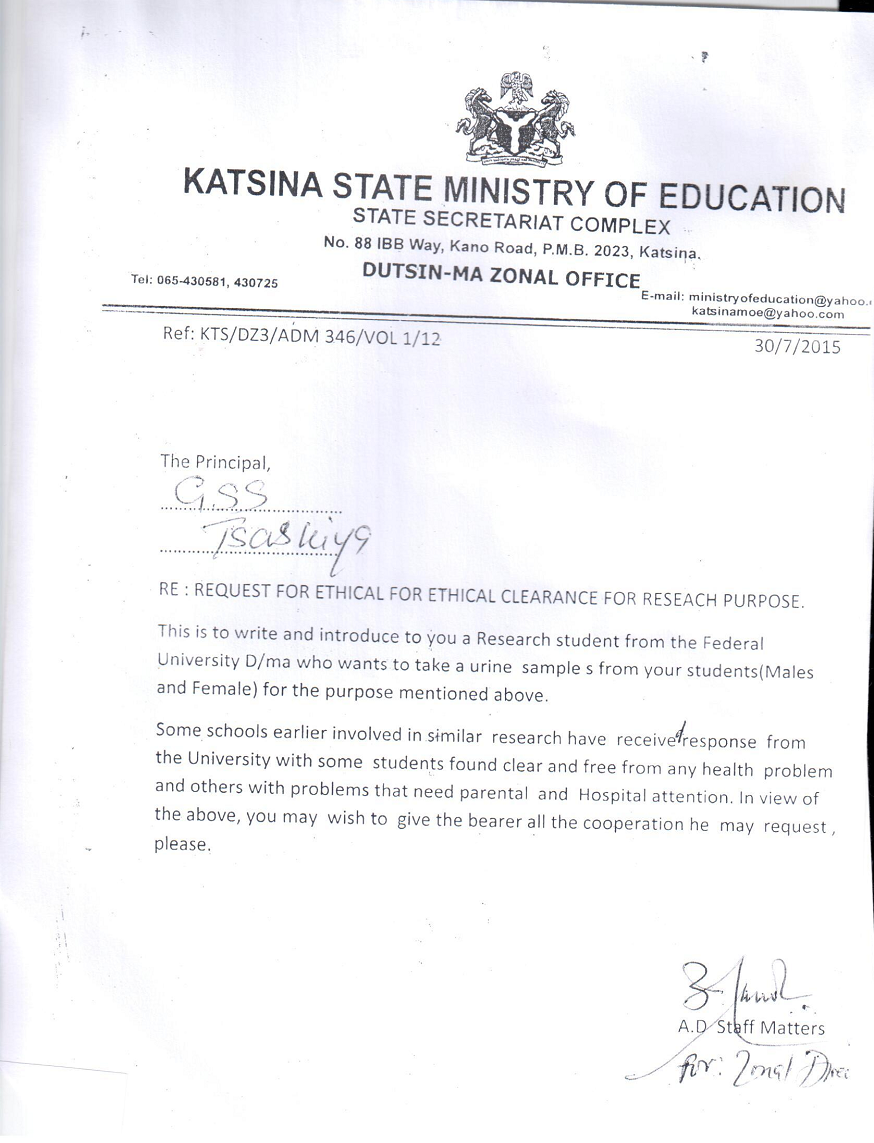


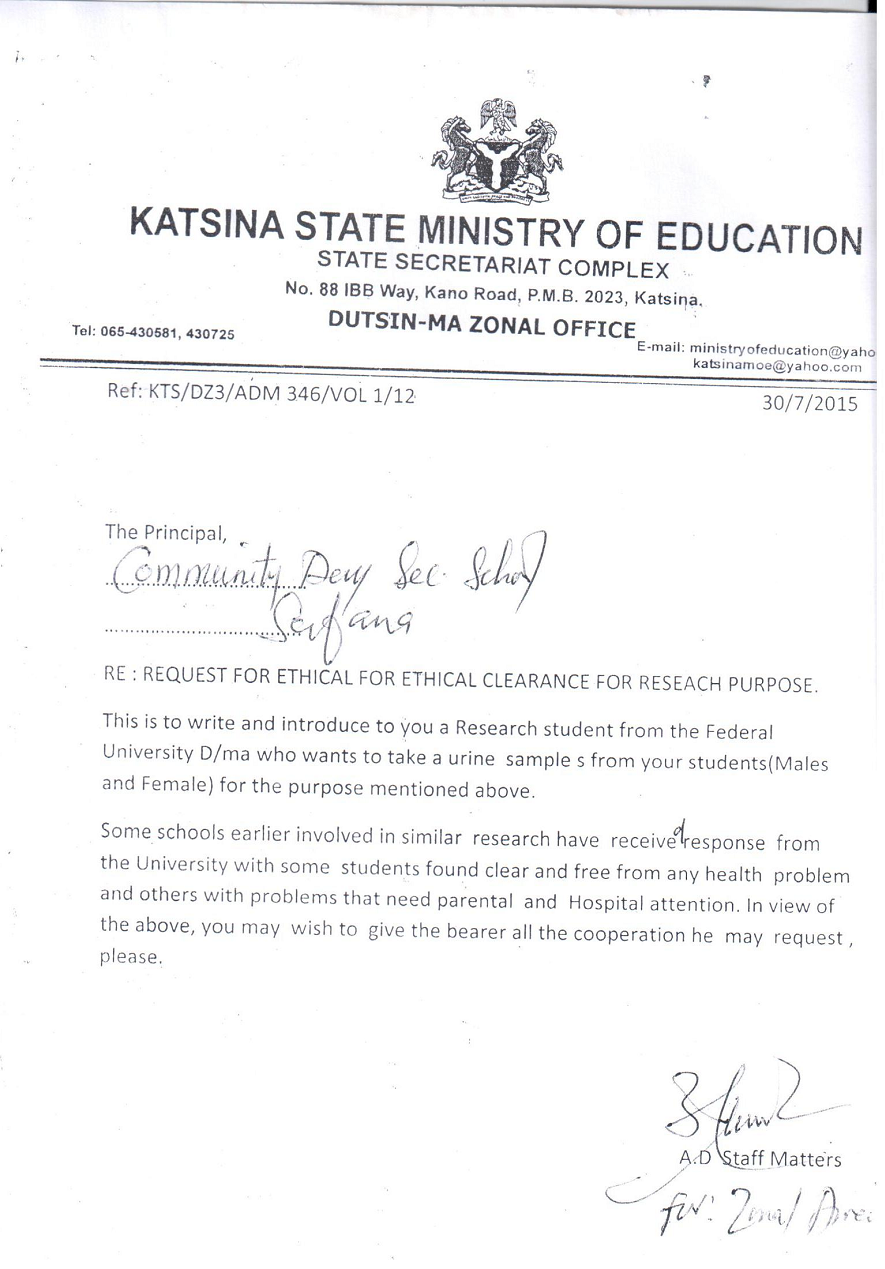


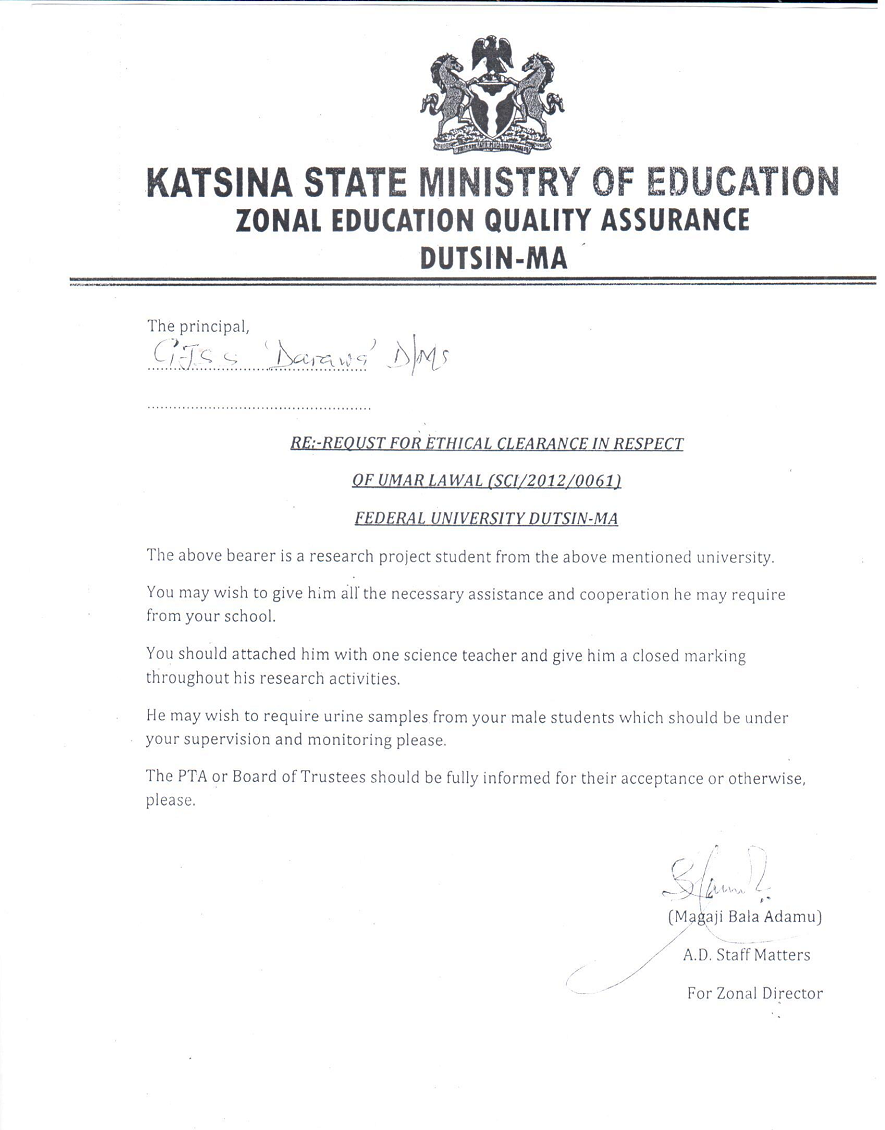


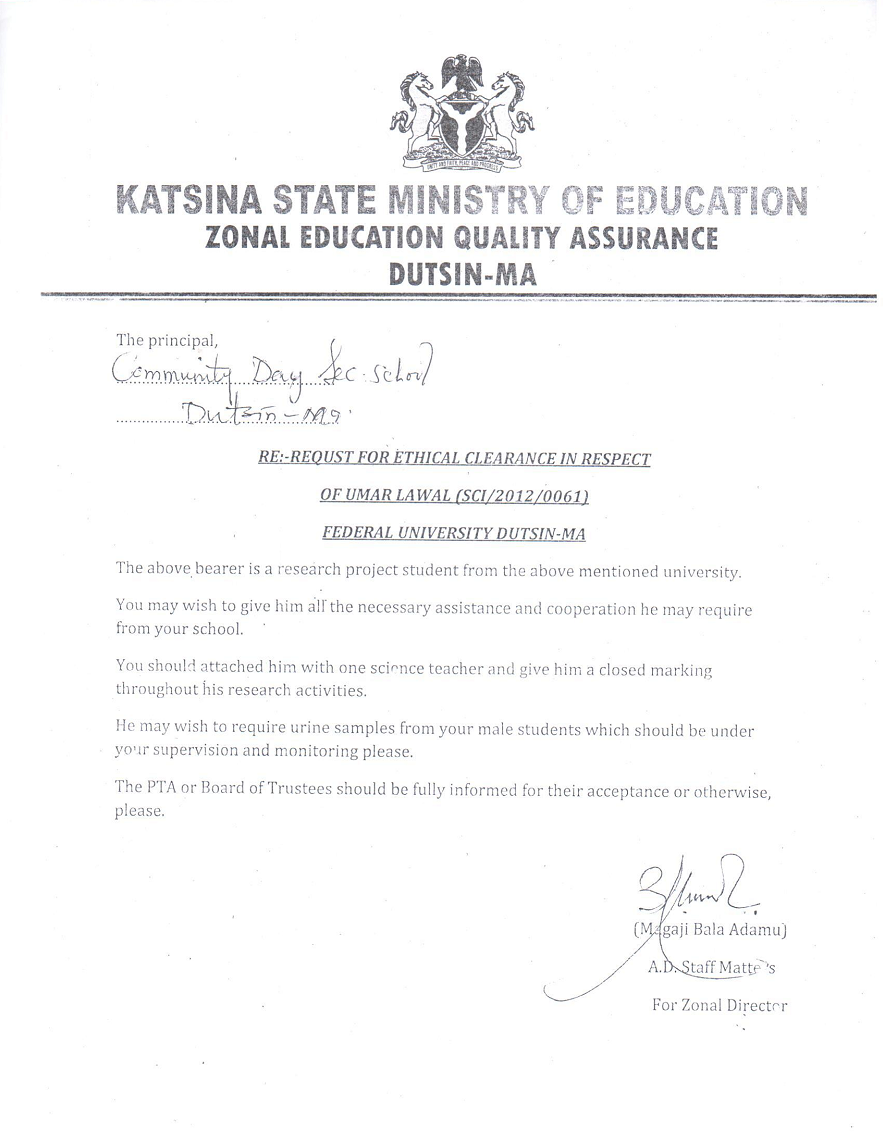


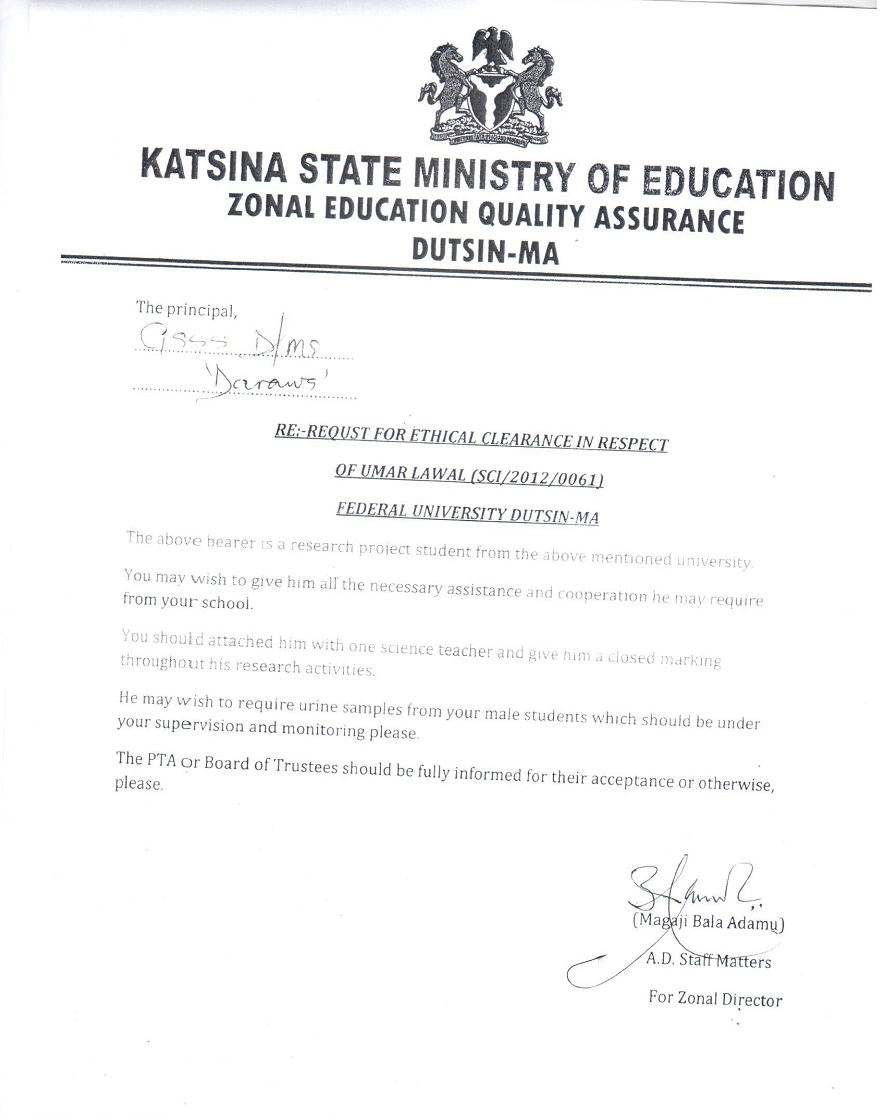

Supplement: S2 File — (DOCX) [file pntd.0005518.s002.docx]
